# Controversies and challenges with COVID-19 in children: Why are they less susceptible and should they be at home rather than in school?

**DOI:** 10.7196/AJTCCM.2020.v26i3.114

**Published:** 2020-09-16

**Authors:** R Masekela, A J Wessels

**Affiliations:** 1 Department of Paediatrics and Child Health, School of Clinical Medicine, College of Health Sciences, University of KwaZulu-Natal, Durban, South Africa; 2 Department of Paediatrics and Child Health, Queen Nandi Regional Hospital, School of Clinical Medicine, College of Health Sciences, University of KwaZulu-Natal, Durban, South Africa

**Keywords:** SARS-cov2, South Africa challenges, Low middle income country, Economy, COVID-19, Children

## Editorial

Severe acute respiratory syndrome coronavirus 2 (SARS-CoV-2) disease, also known as COVID-19, was first identified in 4 cases in China in December 2019.^[1]^ The first case in South Africa (SA) was identified in a person returning from a holiday in Italy on 5 March 2020. As of 3 September 2020, SA has more than 600 000 confirmed SARS-CoV-2 cases and 8 366 deaths.^[2]^ SA decided, as with most countries globally, to close schools once the first few cases of SARS-CoV-2 had been identified in the country, on 16 March 2020.^[3]^ This was followed by a phased approach of reopening the economy and schools with two grades (7 and 12) on 8 June 2020. This decision was made in the face of increasing SARS-CoV-2 infections around the country prior to the peak in all of the nine provinces. The honourable President Cyril Ramaphosa then announced the closure of SA public schools on 23 July 2020, citing the surge in infections as the basis of the recommendation of school closures.^[4]^

The question therefore remains as to what the scientific basis is for keeping schools open or closed during a pandemic. Current data available from the National Institute of Communicable study on children show that although there are over 20 million children in SA <18 years old, constituting 35% of the population, they only contribute 6% of SARS-CoV-2 confirmed cases.^[5]^ Of the total population of admissions to hospital, children only constituted 3.3% of all admissions, and of these, the majority were children <3 years (i.e. pre-schoolers). Of the children who required admission, only 2.4% required ventilation in the intensive care unit. Although the highest incidence risk is in the age group 15 - 18 years, adolescents have a lower proportion of admissions.^[5]^ A number of societies, including the SA Paediatric Association and the Paediatric Management Group, have released statements on the scientific basis for keeping schools open.^[6]^

The scientific basis for the argument of the paediatric organisations was based on five pillars unique to children:^[7–11]^

(i) lower risk of children in acquiring SARS-CoV-2
(ii) lower transmission risk of children to adults and other children
(iii) lower comorbidities profile in children
(iv) very low risk of death
(v) collateral damage of school closures in the SA context.

So, what makes SA unique such that we should take what some consider a radical approach in keeping schools open going into a peak in a pandemic? SA remains a highly unequal society where 25 years post democracy almost half of all children (over 9.1 million) still depend on school feeding schemes to get at least one nutritious meal per day.^[12]^ A further 2.5 - 3.5 million children aged 3 to 5 years attend early childhood development centres, where many receive meals.^[13]^ If schools remain closed, these children will not have access to these meals, and malnutrition rates will go up. This is in the face of a pandemic that has had a catastrophic impact on the economy of the country, with huge job losses. Unemployment has already increased by 1 percentage point when comparing the first quarter of 2020 with the last quarter of 2019.^[14]^ Many families with small children are employed in the informal sector and are particularly vulnerable at this time, being unable to access formal relief measures.^[15]^

Home-based learning will further worsen the inequity of education in our country. Effective home-based learning requires technology, internet access and affordable data. In the lower grades and foundation phase, it also requires adult input from educators or attentive, well-educated caregivers. In SA, only 60% of the population have access to internet, and the majority of this access is via mobile phone networks. Under 10% have access to home-installed internet, with as little as 1.6% in rural areas.^[16]^ The most vulnerable children, therefore, have little access to the required technology, and may not have access to a caregiver able to continue with the work at home – either because of age, education level, caring for multiple children, household tasks or employment taking them out of the home.

Almost half of all children in SA are from single-parent households,^[17]^ resulting in lack of supervision as parents return to work with the economy opening up. This will increase the risk of injuries, poisoning and possible abuse related to lack of supervision and safeguarding.

Prolonged school closure has been shown to increase anxiety and depression in children.^[18]^ In SA, call volumes to children’s helplines increased substantially over the first part of the nationwide lockdown, with health-related questions and fears increasing by 99.6% and calls about psychological health increasing by 85%.^[19]^

The counter-argument for keeping schools closed has been concern about increased risk of infections, cluster outbreaks and fear of spread of infection to adults, including teachers and support staff who may be more vulnerable. The lack of adequate infrastructure, which includes large class sizes, lack of running water and ablution facilities, forms part of the debate on the challenges on opening schools.

The unique context in SA, where the majority of children come from multigenerational households where caregivers may be at high risk, especially where elderly grandmothers are the primary caregivers, should also be considered. Living with individuals with comorbidities such as HIV, which has an adjusted hazard ratio for death of 1.78 (95% confidence interval 1.38 - 2.29) irrespective of viral suppression,^[20]^ increases this risk.

Our conclusion is that while we are all learning about SARS-CoV-2, based on the above arguments and our unique SA context, schools should remain open in order for the majority of children in this country to benefit from a safer learning environment, nutrition and mental health, and from their right to learning in the face of a pandemic that largely affects adults, but will have lasting and irreparable effects on their educational and developmental needs.
